# The Prevalence of Temporomandibular Disorders Among Dental and Medical Students in Taif, Saudi Arabia: A Cross-Sectional Study

**DOI:** 10.7759/cureus.65827

**Published:** 2024-07-31

**Authors:** Abdulaziz A Alharbi, Bandar S Shukr, Murayziq A Algethami, Faris Y Alhumaidi, Nawaf M Mohaymidan

**Affiliations:** 1 Department of Oral and Maxillofacial Surgery and Diagnostic Sciences, Faculty of Dentistry, Taif University, Taif, SAU; 2 Department of Preventive Dentistry, Faculty of Dentistry, Taif University, Taif, SAU; 3 Department of Dentistry, Faculty of Dentistry, Taif University, Taif, SAU

**Keywords:** temporomandibular disorder (tmd), prevalence, healthcare students, helkimo index, temporomandibular joint-tmj

## Abstract

Objectives

This study was undertaken to investigate the prevalence of temporomandibular disorders (TMDs) and the impact of various contributing factors among undergraduate healthcare students in the region of Taif, Saudi Arabia.

Methods

A total of 100 undergraduate students were recruited from both the College of Medicine and the College of Dentistry at Taif University, Taif, Saudi Arabia. Data were collected on demographic parameters and the Helkimo’s index (anamnestic {Ai} and clinical dysfunction {Di} component) using an anonymous self-administered questionnaire, as well as clinical examinations.

Results

A significantly high prevalence (97%) of TMDs was observed among the sampled students, with most of them (44.0%) experiencing severe symptoms that might negatively impact their quality of life. On clinical examinations, most of the students (75.0%) showed signs of mild clinical dysfunction, which might indicate an early stage of TMD. Moreover, factors that include older age, majoring in “dentistry” studies, being allergic, having oral habits, poor mental health, and previous COVID-19 infections were found to be significantly associated with TMDs.

Conclusion

The findings indicate a relatively high TMD prevalence among the sampled undergraduate healthcare students, especially those studying “dentistry”. Curriculum modifications, coupled with more awareness and education, are recommended to achieve early diagnosis and help in reducing the incidence of TMD among this population.

## Introduction

Temporomandibular disorder (TMD) is a broad term that refers to a variety of disorders affecting the temporomandibular joint (TMJ), masticatory muscles, and occlusion, with symptoms including pain and discomfort, limited jaw mobility, muscular tenderness, and frequent joint noises [1]. TMD can also be known as ‘any disorder that involves discomfort and/or dysfunction of the TMJ and the surrounding structures’ [2,3]. TMD was first studied in the 1950s [4]. At that time, it was believed that incorrect occlusion was the primary cause of TMD, affecting the mobility of the muscles of mastication [4]. However, during the 1960s and the 1970s, both emotional stress and occlusal abnormalities were considered the main causative factors of TMDs [4]. Nowadays, with further TMD research, it is well-established that TMDs are multi-factorial in origin, arising from several factors, such as emotional stress, occlusal abnormalities, and interferences, tooth loss, dysfunction of masticator muscles, deviations in the posture, and changes related to TMJ structure either internally or externally, as well as various associations of these causative factors [4]. 

Patients with TMDs can experience one or more of the following symptoms: clicking sound in the TMJ area, a feeling of fatigue in the jaw area, stiffness of the jaw when waking up or during mouth opening, mandibular locking or luxation during mouth opening, pain or discomfort when opening the mouth, and pain in the TMJ area or the in the muscles of mastication [5]. TMDs can be diagnosed by the presence of a limited range of mandibular movement, reduced TMJ function, or tenderness, either in the TMJ structure, the masticator muscles, or during mouth opening [5]. Some studies reported that the signs and symptoms of TMDs increase as people get older [6]. Other studies, on the contrary, have found that as people get older, their symptoms lessen [6].

Since there are no standardized guidelines for estimating the severity of TMDs, indices are crucial tools for determining the severity of the condition in a certain group of people [7]. Helkimo was one of the first scientists to develop indicators to assess the prevalence and severity of TMDs [7]. He established the Helkimo index in an attempt to measure the severity of TMDs in any population using a standard method [5]. Research indicates that TMDs affect 25% of the population, however, only a small portion of people require therapy for TMDs [8,9]. Moreover, the prevalence of TMD symptoms is notably higher in women, with the most affected age group ranging between 20 years and 50 years [8,9].

TMDs are a significant concern among students in the healthcare field, likely due to the high demands and stress associated with their academic studies. The recent literature underscores the necessity for sufficient research to accurately understand the prevalence and determinants of TMDs among healthcare students, particularly in the population of Saudi Arabia. The prevalence of TMDs among healthcare students in Saudi Arabia varies. In a study conducted on university students in Riyadh, which may include medical students, the prevalence of TMDs was reported to range between 1.1% and 36.1% [10]. Another study reported a prevalence of almost 37% among dental students [11]. The most commonly reported symptoms by the participants of this study were pain arising from the jaw, temple, and peri-auricular area [11]. Moreover, factors such as being female, married, and being in clinical academic levels were significantly associated with a higher risk of TMDs [11]. Psychosocial factors, such as anxiety and parafunctional habits, were also found to be linked to an increased risk of developing TMDs among the participants [11]. These studies highlight the importance of raising awareness and advocating for preventive strategies for TMDs among healthcare students, who may be particularly vulnerable due to the demands and stress of their education and future profession. The current literature lacks evidence about TMDs among students of healthcare-related fields in Taif, Saudi Arabia. Therefore, this study was undertaken to provide a nuanced view regarding TMDs and related factors via the use of Helkimo’s index (anamnestic {Ai} and clinical dysfunction {Di} component) among a population of healthcare students (medical and dental) in the city of Taif, Saudi Arabia.

## Materials and methods

Study design and ethical considerations

This cross‑sectional observational study was conducted between September 2022 and March 2023 to investigate the distribution of TMDs among a sample of non-patient population (healthcare students) attending Taif University, Taif, Saudi Arabia. The study protocol was reviewed and approved by the institution’s ethical research committee (IRB no: 43-139). All participants were enrolled in the study after obtaining written informed consent. The manuscript was prepared by adhering to the guidelines of the Strengthening the Reporting of Observational Studies in Epidemiology (STROBE) [12].

Study sample

At the time of the data collection, the total number of students in all academic levels, in both the College of Medicine and College of Dentistry at Taif University, was estimated to be 310. The "Raoasft" website was used to calculate the sample size, which was determined to be at least 172 individuals, with a 5% margin of error and a 95% confidence range. Nevertheless, to generate reliable estimations, a total of 180 students were invited to participate in the study. However, only 122 male students agreed to participate and provided written informed consent. The students were recruited using a convenient sampling method.

Inclusion and Exclusion Criteria

The inclusion criteria were undergraduate medical or dental students with all permanent teeth, as well as the absence of any history of orthodontic treatments. The exclusion criteria included non-medical and non-dental students, students with missing permanent teeth (except the third molar), students with a previous history of orthodontic treatments, as well as students with a previous diagnosis of either a TMD, a stomatognathic system impairment, or a pathological condition in the ear (n = 22). 

Data collection

Data were gathered by an anonymous self-administered questionnaire that was adapted to the current study's specifications from previously validated studies [13-19]. The questionnaire was modified by adding more demographic questions, as well as specifying the location (unilateral or bilateral) of some items in the anamnestic component (Ai) list. The questionnaire was administered to the participants, together with a cover letter outlining the objectives, potential risks, and benefits of the study. The study questionnaire consisted of three sections. Data on demographics, past medical history, seeing a psychiatrist, and oral habits (e.g., bruxism, nuts eating, and nail biting) were collected in the first section. In addition, the participants were asked if they had been previously infected with COVID-19. The next two sections were used to collect the subjective symptoms of the Helkimo index [5].

One section was used for collecting data about the anamnestic component (Ai) of the index, which involved eight questions that focused on symptoms reported by the students (Table 1). The students had to answer either “yes” or “no” to each question. Some "yes" responses were given an additional description of either a “unilateral” or “bilateral” incident. Student responses then were categorized using the following scale: Ai0 (no symptoms), AiI (mild symptoms included sensation of the jaw fatigue, jaw stiffness, and TMJ sounds {clicking or crepitus}), AiII (severe symptoms included one or more of the following: {a} difficulty in the mouth opening, {b} jaw locking, {c} mandible dislocation and its painful movement, and {d} painful TMJ region and/or masticatory muscles) [7,18]. 

**Table 1:** Assessment of the anamnestic component (Ai) of the Helkimo index. TMJ: temporomandibular joint

Question	Answer			
Do you have a sound (clicking or crepitation) in the area of the TMJ?	Yes “unilateral”		Yes “bilateral”	No
Do you feel fatigued in the jaw area?	Yes “unilateral”	Yes “bilateral”		No
Do you have pain in the TMJ or the area of the masticatory muscles?	Yes “unilateral”	Yes “bilateral”		No
Do you have pain during movement of the mandible?	Yes “unilateral”	Yes “bilateral”		No
Do you have difficulty opening your mouth?	Yes			No
Do you have a locked mandible during opening the mouth?	Yes			No
Do you have luxation of the mandible?	Yes			No
Do you have jaw rigidity during awakening or slow movement of the mandible?	Yes			No

The last section involved a clinical examination to record the clinical dysfunction component (Di) of the index. For this, a modified version of Helkimo’s dysfunction index (Di) was used. The clinical examination involved the assessment of five criteria: opening of the mandible, deviation during opening, dysfunction of the TMJ, pain in the TMJ or the surrounding region, and tenderness of the masticatory muscles during palpation [7]. The mandibular opening was determined by asking the participant to gently open the mouth and measuring the distance between the maxillary and the mandibular central incisors using a ruler [7]. Deviation during opening was evaluated by asking the participant to gently open the mouth and observing any deviations from the midline between the upper and the lower arch [7]. For TMJ dysfunction, the TMJ was assessed for the presence of clicking, locking, or luxation, without the use of the stethoscope [7]. Finally, a bilateral palpation was performed to record any pain or tenderness in the TMJ and the preauricular area, as well as in the muscles of mastication [7]. Scores assigned for the clinical dysfunction (Di) component are shown in Table 2. The total score was calculated by summing up the scores of all five elements [7]. The range of the total score was between 0 and 25, with higher scores indicating severe/serious condition [7]. Based on the total score, all the participants were categorized as follows: Di0, no dysfunction; DiI, mild dysfunction (score 1-4); DiII, moderate dysfunction (score 5-9); DiIII, severe dysfunction (score 10-25) [7]. Lastly, to minimize the risk of inherent errors, all the clinical examinations were performed by one well-trained examiner who was also calibrated in the use of this index. Before conducting the required examinations, the examiner received theoretical and practical training sessions from an expert in the field for a period of one week, coupled with a final assessment at the end.

**Table 2:** Assessment of the clinical dysfunction component (Di) of the Helkimo index. TMJ: temporomandibular joint

Clinical examination	Score
Mandibular opening	
> 40 mm	0
30-39 mm	1
< 30 mm	5
Mandibular deviation	
< 2 mm	0
2-5 mm	1
> 5 mm	5
TMJ dysfunction	
No impairment	0
Palpable clicking	1
Evident clicking, locking, and luxation	5
TMJ pain	
No pain	0
Palpable pain	1
Palpebral reflex	5
Muscle pain	
No pain	0
Palpable pain	1
Palpebral reflex	5

Statistical analysis

Descriptive statistics were computed for all variables, including means and standard deviations for continuous variables, and frequencies and percentages for categorical ones. For categorical variables, the chi-square test or the non-parametric alternative of Fisher’s exact test was used as appropriate. The data were analyzed using the SPSS software (version 28) (Armonk, NY: IBM Corp.). All tests were two-tailed and a p-value of 0.05 or less was considered statistically significant.

## Results

The study sample consisted of 100 college students with an equal distribution between the Colleges of Dentistry and Medicine. The mean age of participants was 22.30 years with a standard deviation of 2.62 years. A small fraction of the participants reported having a systematic disease (2%) or allergies (5%). Oral habits such as bruxism, nuts eating, and nail-biting were reported by 24% of the students. Phycological factors represented by consultations with a psychiatrist were noted in 15% of the participants. Interestingly, 31% reported having been infected with COVID-19, reflecting the widespread impact of the pandemic among the student population (Table 3).

**Table 3:** Characteristics of the study sample.

Characteristic	Total n = 100
Age (years), mean ± SD	22.30 ± 2.62
College	
Dentistry, n (%)	50 (50.0)
Medicine, n (%)	50 (50.0)
Systematic disease	
Yes, n (%)	2 (2.0)
No, n (%)	98 (98.0)
Allergy	
Yes, n (%)	5 (5.0)
No, n (%)	95 (95.0)
Oral habits (e.g., bruxism, nuts eating, and nail-biting)	
Yes, n (%)	24 (24.0)
No, n (%)	76 (76.0)
Seeing a psychiatrist	
Yes, n (%)	15 (15.0)
No, n (%)	85 (85.0)
Infected with COVID-19	
Yes, n (%)	31 (31.0)
No, n (%)	69 (69.0)

**Figure 1:**
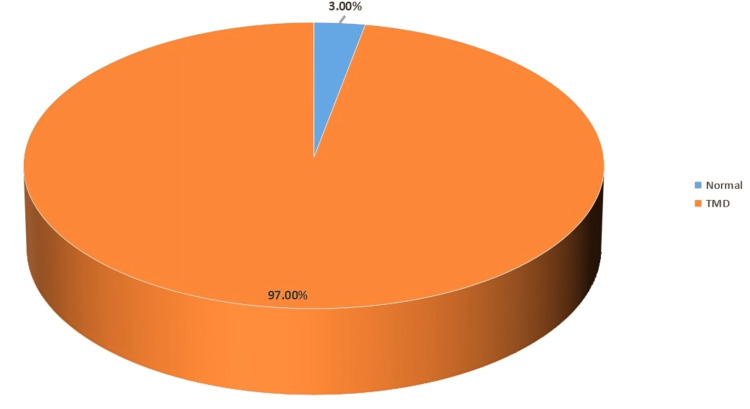
Prevalence of TMDs among the study sample. TMDs: temporomandibular disorders

The findings revealed a significantly high prevalence of TMDs among the sampled population, with a small fraction, precisely 3% of the participants, exhibiting no signs or symptoms of TMDs, while a significant majority, amounting to 97% of the participants, had one or more of the signs and symptoms of TMDs (Figure 1). The analysis of the anamnestic and clinical dysfunction components revealed a notable distribution of TMD symptoms and dysfunctions among the students (Table 4). Around 17.0% of the students experienced mild symptoms, suggesting a low impact of TMD on daily activities. Additionally, a larger proportion (44.0%) were affected by severe symptoms, which may significantly interfere with quality of life. Moreover, most of the participants (75.0%) exhibited mild clinical dysfunction, potentially indicating early stages of TMD, while 16.0% presented with moderate dysfunction, requiring clinical attention. Only a small subset (3.0%) of the students suffered from severe dysfunction, necessitating immediate and comprehensive interventions.

**Table 4:** Assessment of Helkimo index components among the study samples (n = 100).

Component	n (%)
Anamnestic component (Ai)	
Ai0 (free of symptoms)	39 (39.0)
AiI (mild symptoms)	17 (17.0)
AiII (severe symptoms)	44 (44.0)
Clinical dysfunction component (Di)	
Di0 (no dysfunction)	6 (6.0)
DiI (mild dysfunction)	75 (75.0)
DiII (moderate dysfunction)	16 (16.0)
DiIII (severe dysfunction)	3 (3.0)

The relations between demographics and Helkimo index components were also evaluated in the sampled students (Table 5). Regarding age, milder forms of TMD were found to be prevalent (31.1%) among young students who were 22 years of age or less, while those who were 23 years or older exhibited a higher prevalence (53.8%) of severe TMD symptoms (p = 0.001). This was also reflected in the clinical dysfunction component, with mild dysfunction being higher (85.4%) in young students, while moderate (23.1%) to severe (5.8%) dysfunctions were prevalent among older students. However, this finding was borderline significant (p = 0.053). A significant difference was also observed based on the field of healthcare study. Medicine students showed higher rates of mild TMD symptoms (30.0%) and clinical dysfunction (90.0%), while dentistry students exhibited severe symptoms (48.0%), as well as moderate (28.0%) to severe (6.0%) dysfunctions (p-value for Ai = 0.002 and p-value for Di = 0.001, respectively). No significant association was found between systematic disease and the Helkimo index components. However, participants with allergies showed a higher prevalence of mild TMD symptoms (60.0%; p = 0.026).

**Table 5:** Associations between demographics and Helkimo index components (n = 100). *P-value ≤ 0.05 was considered statistically significant.

Variable	Anamnestic component (Ai)				Clinical dysfunction component (Di)				
	Ai0 (no symptoms), n (%)	AiI (mild symptoms), n (%)	AiII (severe symptoms), n (%)	p-Value	Di0 (no dysfunction), n (%)	DiI (mild dysfunction), n (%)	DiII (moderate dysfunction), n (%)	DiIII (severe dysfunction), n (%)	p-Value
Age									
22 years or less	17 (35.6)	15 (31.1)	16 (33.3)	0.001*	3 (6.3)	41 (85.4)	4 (8.3)	0 (0.0)	0.053
23 years or older	22 (42.4)	2 (3.8)	28 (53.8)		3 (5.7)	34 (65.4)	12 (23.1)	3 (5.8)	
Collage									
Dentistry	24 (48.0)	2 (4.0)	24 (48.0)	0.002*	3 (6.0)	30 (60.0)	14 (28.0)	3 (6.0)	0.001*
Medicine	15 (30.0)	15 (30.0)	20 (40.0)		3 (6.0)	45 (90.0)	2 (4.0)	0 (0.0)	
Systematic disease									
Yes	0 (0.0)	1 (50.0)	1 (50.0)	0.462	0 (0.0)	2 (100.0)	0 (0.0)	0 (0.0)	1.00
No	39 (39.8)	16 (16.3)	43 (43.9)		6 (6.1)	73 (74.5)	16 (16.3)	3 (3.1)	
Allergy									
Yes	0 (0.0)	3 (60.0)	2 (40.0)	0.026*	1 (20.0)	4 (80.0)	0 (0.0)	0 (0.0)	0.405
No	39 (41.1)	14 (14.7)	42 (44.2)		5 (5.3)	71 (74.7)	16 (16.8)	3 (3.2)	
Oral habits									
Yes	4 (16.6)	7 (29.2)	13 (54.2)	0.021*	1 (4.1)	22 (91.7)	1 (4.2)	0 (0.0)	0.165
No	36 (46.0)	10 (13.2)	31 (40.8)		5 (6.7)	53 (69.7)	15 (19.7)	3 (3.9)	
Seeing a psychiatrist									
Yes	1 (6.7)	6 (40.0)	8 (53.3)	0.005*	1 (6.7)	14 (93.3)	0 (0.0)	0 (0.0)	0.210
No	38 (44.7)	11 (12.9)	36 (42.4)		5 (5.9)	61 (71.8)	16 (18.8)	3 (3.5)	
Infected with COVID-19									
Yes	7 (22.6)	8 (25.8)	16 (51.6)	0.058	2 (6.4)	22 (71.0)	6 (19.4)	1 (3.2)	0.902
No	32 (46.4)	9 (13.0)	28 (40.6)		4 (5.8)	53 (76.8)	10 (14.5)	2 (2.9)	

Regarding oral habits and mental health, those with oral habits significantly suffered from TMD symptoms in both mild (29.2%) and severe (54.2%) forms (p = 0.021). Likewise, those with psychological problems and currently seeing a psychiatrist had a higher prevalence of mild (40.0%), as well as severe (53.3%) TMD symptoms (p = 0.005). Finally, participants who were infected with COVID-19 displayed a higher rate of TMD symptoms in both mild (25.8%) and severe (51.6%) forms, however, this observation was of borderline significance (p = 0.058).

## Discussion

Research on temporomandibular disorders (TMDs) among healthcare students in Saudi Arabia has recently gained increasing interest due to the substantial impact of these conditions on students' quality of life and academic performance. With a few TMD investigations conducted in the country of Saudi Arabia, particularly in the Taif region, the present study is unique in exploring the distribution of TMDs and the impact of various contributing factors among healthcare students in the region of Taif, Saudi Arabia. The findings revealed a significantly high prevalence of TMDs among the sampled students, with most of them experiencing severe symptoms that might negatively impact their quality of life. Additionally, on clinical examinations, more than half of the participants showed signs of mild clinical dysfunction, which might indicate an early stage of TMD. Moreover, factors that include age, field of healthcare study, allergy, oral habits, poor mental health, and COVID-19 infection were found to be significantly associated with TMDs.

In this study, 97% of the participants were found to have some signs and symptoms of TMD. This prevalence was much higher than the prevalence reported by the study of Srivastava et al. that was conducted on a non-patient population (dental students) in Saudi Arabia, which showed a prevalence rate of almost 37% [11]. Similarly, the prevalence was found to be higher compared to the prevalence reported by Nadershah in his study of adult patients in Jeddah, Saudi Arabia, which was found to be 35% [20]. Additionally, the prevalence was greater than the one reported by a study of undergraduate students at Taibah University (53.3%) [21], as well as greater than the study of undergraduate medical, dental, and pharmacy students in Dammam, Saudi Arabia (54.2%) [22]. The variations in TMD prevalence could possibly be due to the lack of standardization, as well as the use of various assessment tools in these studies, with some of them not including any clinical examinations (i.e., TMD/pain screener questionnaire) [20].

TMDs can manifest in different symptoms, most of which can be identified using the anamnestic component (Ai) of the Helkimo index. In this study, a sound (clicking or crepitation) in the TMJ area was the most common symptom reported by the study participants (45% in total: 38% bilateral and 7% unilateral). This was consistent with a study by Rani et al. that was conducted on dental students in India [7], as well as another study done by Gopal et al. on a population of Indian dental patients [18]. The second most common reported symptom was pain in the TMJ or in the muscles of mastication (36% in total: 30% bilateral and 6% unilateral). This finding was in line with the study of Saudi dental students done by Srivastava et al. [11], as well as the study of Saudi adult patients conducted by Nadershah [20]. Regarding clinical examinations, the findings showed that most of the affected students had limited mouth opening (79%), followed by TMJ dysfunction in the form of clicking or crepitus sounds (42%). This could not be compared with the findings from other research conducted on Saudi populations because of the use of different clinical assessment criteria for TMDs.

The present study investigated the possible associations of TMD with different demographic parameters. The findings revealed that older age could be a risk factor for TMD, as a severe form of TMD was significantly more prevalent in older students (23 years or older) than younger ones. Surprisingly, the current evidence regarding age is mixed, with some studies highlighting older age (until middle age) as a significant risk factor [23,24], while others found TMD predominant in younger populations [25,26]. Additionally, age was not a significant contributor to the TMD studies conducted in the Saudi population [11,21,22]. The field of healthcare study was also found to be significantly associated with TMDs, with moderate to severe forms of TMD being prevalent among dental students compared to students studying medicine. The intricate nature of the curriculum and the intensive method of study practiced in Saudi dental schools/colleges are likely the causes for such a pattern in addition to the increased awareness about the symptoms and indicators of TMD among the participants from the dental college [11]. Zafar et al. noticed a similar finding in their study of Saudi students [21] that reported higher rates of TMD among dental students than pharmacy and medical students. In contrast, another study in Saudi Arabia reported a higher prevalence of TMD among undergraduate pharmacy students than dental students [22]. 

The present study did not find any evidence of a significant association between TMDs and systematic illness. This may be because of the small number (2%) of students who reported having a systemic condition. However, allergy was found to be significantly associated with mild TMD symptoms. This finding was in line with another research that found allergy, along with other comorbid conditions, to be common in patients with TMD [27]. This could indicate that allergy might be part of the clinical manifestations associated with TMD. Nevertheless, further interdisciplinary research is needed to better understand the potential links between TMD and systemic illnesses, including allergies, and to develop effective interventions. In a recent systematic review, several factors, such as depression and oral parafunction, were found to have strong relationships with the onset or progression of TMD [28]. Srivastava et al. also reported similar effects in their study of Saudi dental students [11]. Moreover, test anxiety was found to be significantly associated with TMD in the study of undergraduate healthcare Saudi students done by Alamri et al. [22]. These findings were in accordance with the results of the present study that found oral habits and psychological factors to be significantly associated with TMDs in the sampled students. These observations highlight the impact of psychosocial factors on the occurrence of TMDs. The student population has been shown to display higher levels of stress and anxiety compared to other populations due to their continuous exposure to demanding learning environments [29]. Another interesting observation noticed in the present study was the higher prevalence of TMD among students who were previously infected with COVID-19. This was in agreement with another investigation that showed an ongoing adverse effect of the COVID-19 pandemic on TMD symptoms, even after the pandemic had subsided [30]. While direct causation might be difficult to establish, one possible explanation is the elevated levels of stress, anxiety, and depression caused by the pandemic [31,32], which have been demonstrated to be important etiological factors for the development and progression of TMD [28,33]. Nevertheless, further research is likely to provide clear insights into this relationship.

It is clear from the findings of the present study and the discussion of previously relevant research that a considerable percentage of undergraduate healthcare students experienced either TMD or its exacerbating conditions. Early diagnosis and intervention are critical to improve the prognosis of TMD and prevent permanent TMJ damage [34]. However, delayed diagnosis might lead to permanent tissue damage and potentially ankylosis, requiring complex surgical treatment [35]. The diagnosis of TMD can be difficult because of its multiple causative factors and challenging clinical presentation [21]. Hence, clinicians should be more aware of TMD and the different diagnostic methods to identify it at earlier stages. The present study is unique in contributing to the limited body of literature regarding TMDs among undergraduate healthcare students in Saudi Arabia, particularly in the Taif population. The study findings could help in guiding public health initiatives and policymakers to develop awareness programs and policies in an effort to reduce TMD levels among health profession students and affected patients. Despite using a relatively adequate, as well as a diverse sample of students recruited from two different health colleges, the findings of the present study should be interpreted considering a few limitations. First, because there were very few female responses (n = 5) and a high non-response rate, gender differences could not be examined in this study. Therefore, the results may not be generalizable to female healthcare students, and further research is needed to understand TMD prevalence in this sub-population. Second, causal relationships between the different factors and TMD cannot be established due to the cross-sectional nature of the study. Third, the generalizability of the study findings on the general population is not feasible due to the difference in stress levels, mostly driven by the learning process, between the university students and the general population. Fourth, over-reporting of TMD symptoms is expected, especially among dental students, as they are likely to be more aware of TMDs, which could bised the TMD prevalence in this group. Finally, the presence of impacted wisdom teeth was not accounted for, which may influence the study findings, as these teeth might induce TMJ pain and discomfort in some individuals during the eruption process. Nevertheless, the high TMD prevalence observed in the current sample of healthcare students in Taif, Saudi Arabia, necessitates further awareness and education about TMD and its contributing factors for both clinicians and the general public. Longitudinal multi-center studies, with adequate samples and sufficient male-to-female ratio, are needed in the future to better understand TMDs and related factors among undergraduate healthcare student populations. Moreover, future studies should compare TMD patterns in different populations (e.g., students of different paths of education).

## Conclusions

The findings of the present study revealed a relatively high TMD prevalence among the sampled undergraduate healthcare students, with most of them exhibiting severe symptoms and mild clinical dysfunction. Additionally, factors such as older age, study field related to dentistry, having an allergic condition, oral parafunctional habits, and compromised mental health, as well as previous COVID-19 infection were found to be significantly related to TMDs. While the cross-sectional design does not establish causality, these findings suggest a strong association between these factors and TMDs and warrant further investigation to confirm causal relationships. Further educational and awareness campaigns to achieve early diagnosis, along with curriculum modifications, might help in lowering TMD incidence and improving the prognosis among the population of undergraduate university healthcare students. However, it is important to note that the findings may not be directly applicable to other student populations or the general population, and future research should consider larger samples and investigate potential gender differences.
